# Remedy for Catarrh

**Published:** 1883-01

**Authors:** Myra May

**Affiliations:** Baraboo, Wis.


Remedy for Catarrh.—Take burnt alum and put it in water (make it quite strong with alum) and snuff it up in your head. Do so two or three times a day until relieved. Myra May.

